# A Silent Intruder: A Case of Asymptomatic Appendix in a Chronic Right Inguinoscrotal Amyand’s Hernia

**DOI:** 10.7759/cureus.92178

**Published:** 2025-09-12

**Authors:** Abhiram Chadive, Rajesh G Gattani, Sana Ahmed, Bhagyesh Sapkale

**Affiliations:** 1 General Surgery, Jawaharlal Nehru Medical College, Datta Meghe Institute of Higher Education and Research, Wardha, IND; 2 Community Medicine, Jawaharlal Nehru Medical College, Datta Meghe Institute of Higher Education and Research, Wardha, IND; 3 Medicine, Jawaharlal Nehru Medical College, Datta Meghe Institute of Higher Education and Research, Wardha, IND

**Keywords:** appendectomy, asymptomatic hernia, diagnosis, hernioplasty, histopathology

## Abstract

Amyand's hernia is accidentally seen through the surgical exploration of an appendix herniation within an inguinal hernia sac. A 60-year-old patient in the case received medical attention for his painless right inguinoscrotal swelling that had been present for 18 years. Clinical examination of the swelling showed reducible swelling with a positive cough impulse. The preoperative ultrasound showed that omentum existed inside the hernia sac. The surgical procedure showed that an asymptomatic appendix was located in the hernia region, thus confirming an Amyand’s hernia diagnosis. Appendectomy, as well as mesh repair during hernioplasty, was performed. Histopathological analysis also confirmed that the appendix had a typical appearance without signs of inflammation. After being discharged from the hospital, the patient experienced a smooth recovery period requiring regular follow-ups with the healthcare team. The given case of Amyand’s hernia shows that proper diagnosis remains challenging, but one must perform surgical exploration on inguinal hernias since rare findings such as Amyand’s hernia are typically diagnosed intraoperatively. Although it is rare, the diagnosis of such conditions is also essential for appropriate surgical management for improving outcomes in patients.

## Introduction

Inguinoscrotal swelling can be defined as an abnormal bulge of the groin and scrotal area which arises from hernia, hydrocele or varicocele, or infection [1]. The inguinoscrotal swelling exists within all age groups that include newborns to elderly persons [1]. A hydrocele, along with a hernia, forms the basic benign reasons for inguinoscrotal swelling, yet one should be aware of testicular torsion or malignancy as serious possible causes [2,3]. The swelling appears during birth as an inherited condition or begins to develop after birth [1]. Inflammation or infections can be indicated through swellings with varied sizes that alter when someone changes positions and present with pain and redness and fever as symptoms [1]. An Amyand's hernia describes an unusual inguinal hernia condition that consists of an appendix in its sac where hernial swelling can become inflamed or perforated [4]. Acute appendicitis symptoms may exist in the condition but are often mistaken for a regular inguinal hernia diagnosis [4]. Children generally present with this condition and are identified during surgical interventions [4]. Asymptomatic appendix without signs of appendicitis exists within hernial sacs as noted in Amyand's hernia cases [4].

## Case presentation

A 60-year-old man presented to the Outpatient Department (OPD) of a Tertiary Care Hospital in India with swelling in the right inguinoscrotal region for the last 18 years. The patient said that the swelling began insidiously and became bigger as the years went by. He added that the size of the swelling increases when he coughs, walks around, and lifts things, while it reduces upon lying down. Nevertheless, the swelling was not painful despite having been present for several years in the case of the given patient. The patient had no history of nausea and vomiting, head trauma, abdominal pain, fever, or recent weight loss. The patient reported increased urinary frequency but had no other bowel or bladder complaints. The medical history did not reveal complaints of hypertension, diabetes, bronchial asthma, tuberculosis, epilepsy, or coronary artery disease.

**Figure 1:**
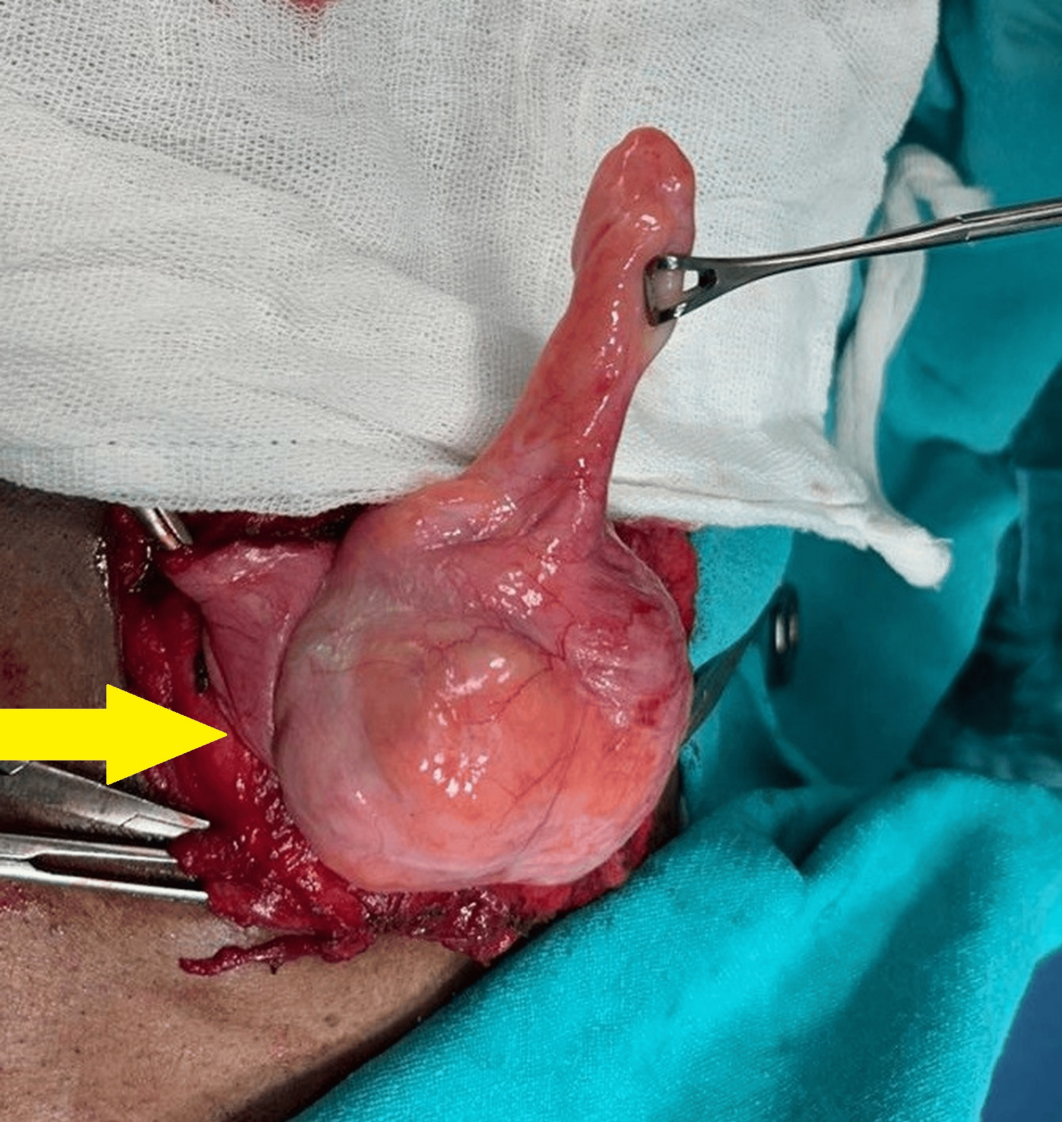
The appendix was discovered as the content of the hernia sac The appendix was observed as content of hernia sac in the given right inguinoscrotal region as highlighted with the yellow coloured arrow.

On general examination, the patient was afebrile with a pulse of 80 beats per minute, a blood pressure of 140/90 mmHg, and a respiratory rate of 18 breaths per minute. No abnormalities were observed on the systemic examination. The respiratory and cardiovascular systems were considered to be normal, and on the aspect of the central nervous system, the findings revealed that the patient was conscious. On local examination, a swelling in the right inguinoscrotal region measuring about 15 x 8 cm in size was observed. The swelling exhibited a positive cough impulse and was reducible in nature. Visible peristalsis was noted within the swelling. There was no evidence of a local rise in temperature, tenderness, or any other groin abnormality. The ultrasound of the patient diagnosed that the content of the hernia sac was greater omentum. Thereafter, the patient was transferred to the operating room, where an open surgical intervention was conducted. On proper dissection, an incision was made in it for exploration, and the sac of hernia was then found. Contrary to ultrasound findings, the appendix was discovered as the content of the hernia sac, confirming the rare diagnosis of Amyand’s hernia as seen in Figure 1.

**Figure 2:**
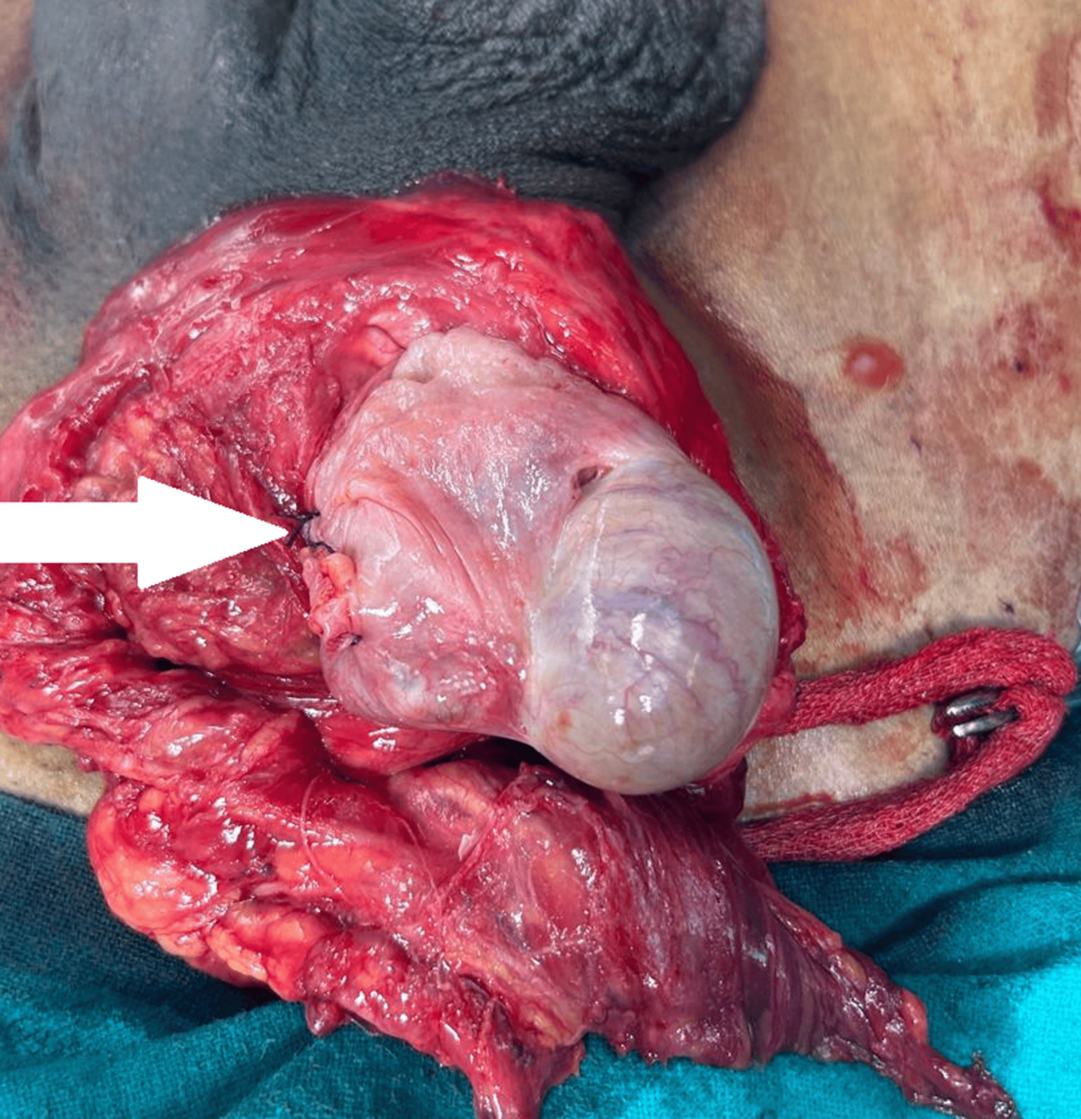
The ligated appendix base using an absorbable suture The appendix base was carefully ligated using an absorbable suture (indicated with white coloured arrow).

The appendectomy was done simultaneously with the repair of Amyand’s hernia during the surgical operation. Once the hernia sac was established to be correctly opened, the appendix, which was found within the sac, was identified and dissected. Standard surgical technique was used, mesoappendix was identified, and then appendiceal artery was meticulously ligated to prevent any further bleeding. The appendix was then tied and cut-off, to minimize the risk of letting luminal contents spill out. The appendix base was carefully ligated using an absorbable suture and then invaginated back into the cecum in order to achieve a proper, tight closure of the structure, as shown in Figure 2.

**Figure 3:**
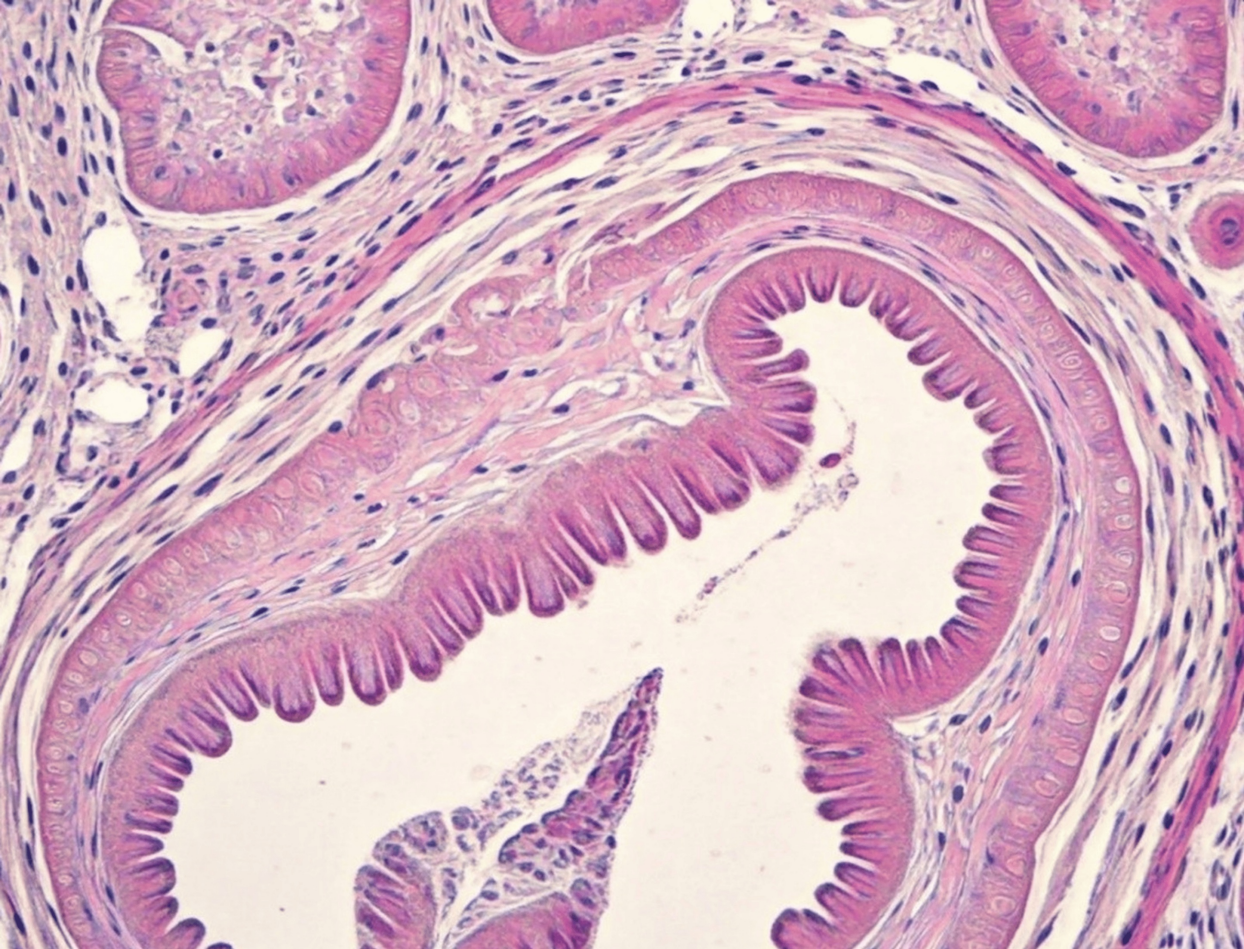
Histopathology image of the appendix confirming diagnosis of Amyand’s hernia Intact appendix observed under microscope as histopathology image with normal mucosa, submucosa, muscularis propria, and serosa layers.

The part of the appendix was then taken out and sent for histopathological analysis. The histopathology image of the appendix confirming diagnosis of Amyand’s hernia is shown in Figure 3. The histopathological image of the resected appendix in the present case showed an intact appendix. The histology revealed retention of architecture with normal mucosa, submucosa, muscularis propria, and serosa. Acute inflammation, ulceration, or perforation was not observed, indicating the absence of appendicitis. Moreover, no neutrophil infiltration, pus cells, or other pathological changes were noted which emphasizes the fact that the appendix was clinically asymptomatic. Slightly increased vascular permeability with congestion in the pulp and oedema of the surrounding connective tissue were observed, which was attributed to mechanical compression within the hernia sac. Adjacent tissue demonstrated the features of chronic inflammation in accordance with the long-standing nature of the hernia. These studies clearly defined the diagnosis of Amyand’s hernia with the asymptomatic appendix.

In the case of emergency appendectomy, attention was turned to hernia repair and hernioplasty for the main ailment after the operation was done. Right inguinal hernioplasty was performed with the use of prosthetic mesh due to the high likelihood of considering recurrence in the inguinal canal. Further, we also proceeded with the eversion of the sac in order to prevent any risk that might be posed by the redundant sac. The surgical field was thoroughly inspected for ensuring proper haemostasis, and then the wound was closed in layers using standard surgical techniques. There were no intraoperative complications during the entire surgical procedure. The patient was further instructed to avoid high-intensity tasks, and a scheduled follow-up was planned in the outpatient department of surgery.

The patient was instructed to come for a follow-up visit after some weeks or months to check on the progress or even emergence of complication or recurrence. The first follow-up was made one week after the surgery to monitor the suture line, look for infection parameters, and confirm proper recovery. Ideally, further follow-ups were expected at one, three, and six months after the operation to determine the prolonged outcome of the hernioplasty, to check whether the deformity had recurred or not, and also to oversee the general health of the patient. During these visits, the patient was instructed to report any signs such as pain, swelling, redness, or discharge at the site of operation. He was further advised to restrict himself from heavy lifting and other rigorous physical activities for about six weeks following the surgery. The patient was advised regarding healthy weight management, along with keeping an appropriate posture to avoid recurrence. The final diagnosis made was of Amyand’s hernia, whereby the content of the hernia was the appendix.

## Discussion

The case described by Khalid et al. was a 28-year-old Pakistani male who developed an indirect right inguinal hernia that stayed asymptomatic for eight years before it became painful and enlarged [5]. Mesh hernioplasty procedures revealed that the appendix existed in the hernia sac, where it displayed minimal congestion alongside the absence of inflammation and severe dense adhesions [5]. Appendectomy, adhesiolysis, and sac excision were performed on the patient [5]. Hospital discharge occurred on the second postoperative day as the patient had an uneventful recovery [5]. In contrast, the presented case was of a 60-year-old male patient with an 18-year progression of a painless right inguinoscrotal hernia. The preoperative ultrasound examination indicated that the omentum existed in this hernia sac, but during surgery, an appendix was found, proving it was an Amyand's hernia. The case by Khalid et al. presented the young patient who needed immediate surgery because of new symptoms, while the older patient needed surgery when Amyand’s hernia was discovered during the operation [5]. Each patient received appendectomy combined with hernioplasty for their procedures [5]. Both cases highlight the value of considering Amyand’s hernia in diagnostic assessments of inguinal hernias and the requirement for surgical examination to achieve proper treatment [5].

The case described by Tsalis et al. was a 60-year-old Greek male who presented with acute right iliac fossa pain and recurrent right inguinal hernia symptoms that were initially suspected to represent another intra-abdominal pathology [6]. A computed tomography (CT) scan showed the patient had an Amyand’s hernia with no signs of inflammation but did show right ureteric lithiasis [6]. The patient underwent a laparoscopic transabdominal preperitoneal (TAPP) hernia repair using polypropylene mesh combined with cholecystectomy surgery, and appendix was found to be normal and left intact [6]. Amyand’s hernia was observed in both cases, yet the diagnosis and management procedures and clinical presentation were dissimilar. A Greek 60-year-old male as described by Tsalis et al. developed acute symptoms that needed intra-abdominal imaging to verify suspected pathology during his treatment, whereas the presented patient showed an ongoing painless hernia whose ultrasound exam misdiagnosed his condition [6]. The laparoscopic surgery on a Greek patient was performed, but the appendix displayed normal findings; hence, it remained untouched in the operation, while the presented case underwent open surgery followed by appendectomy confirmed negative by histopathology analysis [6]. The hernioplasty was performed for both patients successfully, and their postoperative observation confirmed no recurrence of hernia [6].

The case reported by Sadeghi et al. involved a 46-year-old American male who presented with a two-day history of diffuse abdominal pain and a non-reducible, tender right-sided inguinal hernia [7]. CT scan indicated the presence of the appendix inside the hernia sac but showed no signs of inflammation [7]. The diagnosis of the appendix as hernia content occurred in two different ways in the two cases: preoperative imaging detection in the case by Sadeghi et al., and postoperative appendectomy in the presented case because initial ultrasound examination misidentified the hernia contents [7]. The surgical methods used in these cases had several minor variations between them. Tenderness and non-reducibility of the hernia in case reported by Sadeghi et al. led to immediate surgical care, but appendectomy was not required because no inflammation was present [7]. Both cases displayed different conditions, yet they demonstrated the uncommon nature of Amyand’s hernia as well as the need for surgical evaluation during operations to select the most suitable treatment plan [7].

In a case described by Katembo Sikakulya et al., a 40-year-old woman from Uganda had a right-sided non-painful inguinal swelling which showed no change since last one year [8]. The swelling grew larger whenever the patient coughed or lifted heavy objects, although it is not associated with constipation or vomiting [8]. The swelling displayed an ovoid shape and a positive cough impulse while being reducible, non-tender with a size of 3 × 5 cm [8]. The deep ring test was positive, and ultrasound scans showed a right inguinal hernia with a 1.67 cm sized defect, yet the contents of the sac were not specified [8]. The patient presented without any chronic diseases and had no prior surgeries along with normal routine blood test results [8]. The patient received elective herniorrhaphy under local anaesthesia through a treatment with 25 mL of lidocaine 1% mixed with adrenaline [8]. Intraoperatively, a normal appendix was incidentally found within the hernia sac [8]. A modified Bassini technique was used for repairing the hernia after placing the appendix inside the peritoneal cavity [8]. The patient described by Katembo Sikakulya et al. experienced no complication during her recovery period; therefore, she was allowed to leave on the second day [8]. Her day 10 follow-up appointment showed no related complaints which served as a sign of successful surgical outcome [8].

In a case reported by Singh et al., a paediatric patient aged 1.5 years old from India exhibited a persistent left scrotal swelling since birth, which transformed into an irreducible condition in recent times [9]. Bilious vomiting and fever existed with the condition while increasing the suspicion that it involved an obstructed inguinal hernia [9]. A diagnosis of an obstructed left inguinal hernia emerged after clinical examination identified tender swelling that could not be reduced in the left inguinal area [9]. Paediatric patient was stabilized with antibiotics and intravenous fluids before taking him to for surgical intervention [9]. During surgery, the surgical team located the appendix and caecum inside the hernia sac near a perforation that existed alone in the caecum without appendix inflammation [9]. The perforation was localized in the scrotal region and was repaired without the need for peritoneal toileting [9]. Herniotomy included the retention of the appendix for the procedure [9]. The postoperative course was normal, while imaging tests excluded situs inversus, thereby proving that the left-sided Amyand’s hernia developed because of a mobile caecum [9]. The surgical management requires thorough intraoperative analysis to show how different anatomical arrangements affect diagnostic procedures in hernia [9].

In a case reported by Afzal and O'Neill, a 68-year-old female from the United Kingdom was diagnosed with an incarcerated right inguinal hernia following her appearance with sudden right groin pain accompanied by sensitive tissue enlargement [10]. CT scan results confirmed that acute appendicitis existed inside a direct inguinal hernia which is known as an Amyand's hernia [10]. The patient did not show any signs of illness throughout her body examination, and all her blood test results presented normal ranges [10]. During the operation, an open inguinal procedure was performed to find a strangulated distal appendix located inside the hernia sac [10]. During appendectomy, only haemorrhagic fluid was discovered, as well as no signs of purulent contamination inside the hernia sac were present [10]. Bassini darn suture repair was performed instead of mesh to avoid potential postoperative infections [10]. In the case reported by Afzal and O'Neill, the patient was discharged on the following day with oral antibiotics for five-day treatment after she had healed successfully from the operation [10]. Three months following her check-up, she displayed no signs of hernia reappearance [10].

In a case described by Alibrahim et al., a 13-year-old child from Syria was brought to the ambulance service because he showed symptoms including mild fever, stomach pains, and feeling exhausted [11]. His parents dismissed any history of stomach injury during the extended questioning and stated there were no prior medical conditions [11]. During the clinical examination, it was discovered that his stomach muscles were constricted, while abdominal pressure increased his discomfort [11]. His vital signs included temperature, tachycardia, and hyperventilation that remained unpredictable [11]. Laboratory results presented elevated leukocyte levels as a possible sign of inflammatory disease [11]. An abdominal ultrasound test exposed a bloody perfused intestinal loop with hernia along with extrusions and infiltrates surrounding the hernia matter [11]. Urgent surgical intervention became necessary because of the high danger of suffocation [11]. Amyand's hernia was thus diagnosed during surgical exploration due to the presence of an enlarged appendix within the hernia sac [11]. Appendectomy was performed followed by inguinal floor reinforcement during the operation to avoid future hernia occurrences [11]. The patient was provided with 1,000 mg of cephalosporins before discharge to prevent surgical site infection after maintenance for 24 hours [11]. Surgical interventions need early detection of Amyand's hernia because such surgeries require immediate responsiveness toward children presenting with atypical symptoms [11]. Comparative analysis between the case report and reports, which are published in the past is presented in Table 1.

**Table 1:** Comparative analysis between the case report and reports, which are published in the past Comparison of the findings of previous studies with the present study. TAPP: transabdominal preperitoneal, op: operative.

Case	Place/Year	Patient Details	Presentation	Diagnosis Method	Surgical Intervention	Outcome and Clinical Insights
Khalid et al., [5]	Pakistan/2021	28-year-old male	Asymptomatic inguinal hernia for 8 years, later painful and enlarged	Intraoperative discovery	Open mesh hernioplasty with appendectomy	Uneventful recovery, discharged on post-op day 2
Tsalis et al., [6]	Greece/2022	60-year-old male	Acute right iliac fossa pain, recurrent hernia symptoms	CT scan identified Amyand’s hernia, no inflammation	Laparoscopic TAPP hernia repair, appendix left intact	No complications or recurrence post-op
Sadeghi et al., [7]	USA/2024	46-year-old male	2-day history of abdominal pain, non-reducible tender inguinal hernia	CT scan detected appendix in hernia sac, no inflammation	Open surgery, appendectomy not performed	Different pre-op and intra-op findings highlight imaging limitations
Katembo Sikakulya et al., [8].	Uganda/2021	40-year-old female	Right-sided inguinal swelling, non-painful, reducible	Ultrasound confirmed inguinal hernia but did not specify sac content	Herniorrhaphy using Bassini technique, appendix repositioned	Successful surgery, no complications on follow-up
Singh et al., [9]	India/2011	1.5-year-old male	Persistent left scrotal swelling, bilious vomiting, fever	Clinical exam and imaging identified obstructed left inguinal hernia	Herniotomy, appendix, and caecum found inside, perforation repaired	Situs inversus ruled out, left-sided Amyand’s hernia due to mobile caecum
Afzal and O'Neill et al., [10]	UK/2023	68-year-old female	Sudden right groin pain, tender tissue enlargement	CT scan confirmed Amyand’s hernia with acute appendicitis	Open inguinal surgery, appendectomy, Bassini darn suture repair	Discharged on post-op day 1 with antibiotics, no recurrence at 3-month follow-up
Alibrahim et al., [11].	Syria/2023	13-year-old male	Mild fever, abdominal pain, exhaustion	Abdominal ultrasound detected herniated appendix with inflamed bowel	Urgent surgery, appendectomy, inguinal floor reinforcement	Successful intervention, received prophylactic antibiotics, no post-op complications
Present Case	India/2024	60-year-old male	18-year painless right inguinoscrotal hernia, misdiagnosed as omentum content	Ultrasound misidentified content, intraoperative finding confirmed Amyand’s hernia	Open appendectomy and mesh hernioplasty	No recurrence or complications, highlighting need for intraoperative confirmation

## Conclusions

Amyand's hernia, having the appendix within an inguinal hernia sac, is a rare condition that is mostly presented without significant symptoms. Amyand's hernia is often found incidentally during surgical procedures. The presented patient was an elderly male who had a painless right inguinoscrotal hernia that had reduced easily for 18 years. The examination through ultrasound initially misdiagnosed the hernia structure as greater omentum. The appendectomy was performed along with hernioplasty due to the presence of the appendix. Histopathological tests confirmed a normal appendix without inflammation. The given case focuses on the importance of considering Amyand's hernia among other inguinal hernias as a possible diagnosis. Proper medical diagnosis and treatment for inguinal hernias require surgical exploration, as it provides information to manage the condition safely.

## References

[1] Santi M, Lava SA, Simonetti GD, Bianchetti MG, Milani GP (2018). Acute idiopathic scrotal edema: systematic literature review. Eur J Pediatr Surg.

[2] Shakil A, Aparicio K, Barta E, Munez K (2020). Inguinal hernias: diagnosis and management. Am Fam Physician.

[3] Hoang VT, Van HA, Hoang TH, Nguyen TT, Trinh CT (2024). A review of classification, diagnosis, and management of hydrocele. J Ultrasound Med.

[4] Manatakis DK, Tasis N, Antonopoulou MI (2021). Revisiting Amyand's hernia: a 20-year systematic review. World J Surg.

[5] Khalid H, Khan NA, Aziz MA (2021). Amyand's hernia a case report. Int J Surg Case Rep.

[6] Tsalis K, Symeonidis S, Anestiadou E (2022). Amyand's hernia as a random finding in acute abdominal pain and the role of thorough investigation: a case report. Maedica (Bucur).

[7] Sadeghi N, McDermott J, Kermanshahi N, Anasi A, Ahmed I (2024). A case of Amyand's hernia. Cureus.

[8] Katembo Sikakulya F, Kiyaka SM, Masereka R, Onyai P, Anyama P (2021). Incidental discovery of Amyand's hernia in an adult female: a case report. Int J Surg Case Rep.

[9] Singh K, Singh RR, Kaur S (2011). Amyand's hernia. J Indian Assoc Pediatr Surg.

[10] Afzal Z, O'Neill R (2021). Strangulated Amyand's hernia: management during the COVID-19 pandemic. J Surg Case Rep.

[11] Alibrahim H, Albattour M, Swed S, Sawaf B (2021). A strangulated Amyand's hernia: the first case report in Syria. Ann Med Surg (Lond).

